# Role of Radiation Based Conditioning Regimens in Patients With High-Risk AML Undergoing Allogenic Transplantation in Remission or Active Disease and Mechanisms of Post-Transplant Relapse

**DOI:** 10.3389/fonc.2022.802648

**Published:** 2022-02-15

**Authors:** Amandeep Salhotra, Anthony Selwyn Stein

**Affiliations:** Department of Hematology/Hematopoietic Cell Transplant (HCT), City of Hope National Cancer Center, Duarte, CA, United States

**Keywords:** leukemia, HCT, TBI, TMLI, relapse

## Abstract

In the two decades there has been a consistent improvement in the clinical outcomes of patients diagnosed with acute leukemia undergoing allogenic stem cell transplantation. These improvements have been made possible by advancements in supportive care practices, more precise risk stratification of leukemia patients by genetic testing at diagnosis, accurate disease assessment by measurable residual disease (MRD) in pretransplant marrow and attempts to clear residual disease clones prior to transplant. Availability of targeted therapies, immunotherapies, and approval of novel drug combinations with BCL-2 inhibitors has also improved remission rates for patients who are undergoing transplant. For patients who are unable to achieve a morphologic or MRD^-^ remission prior to transplant, the risk of relapse post-transplant remains high. Total body irradiation (TBI) based intensification of transplant conditioning may be able to overcome risk of increased relapse rate in this clinical setting by improving clearance of leukemic clones. However, in the past increased nonrelapse mortality (NRM) associated with escalation of conditioning intensity has neutralized any potential benefit of decreasing relapse rate in HCT patient resulting in no significant improvement in overall survival. In this review we discuss incorporation of newer radiation techniques such as total marrow irradiation (TMI) to safely deliver targeted doses of radiation at higher doses to improve outcomes of patients with active leukemia. We also discuss the mechanisms associated with leukemia relapse and treatment options available in post allo-HCT relapse setting despite use of intensified conditioning regimens.

## 1 Introduction

Allogeneic hematopoietic cell transplantation (HCT) is the only curative option for patients with acute leukemia with high-risk features (1–3). Current guidelines emphasize that HCT should be offered to patients in complete morphologic remission (CR) who are preferably MRD^-^ (4). With contemporary chemotherapy regimens, 25% of patients with Acute Myeloid Leukemia (AML) treated with 7 + 3 induction chemotherapy, and 10-15% of adult patients with Acute Lymphoblastic Leukemia (ALL) treated with HyperCVAD or AYA regimens fail to achieve morphologic remission (5, 6). For these patients with induction-failure or those who relapse after initial remission have very poor prognosis unless remission can be obtained with salvage regimens for HCT to be performed (7, 8). Allo-HCT in patients with active disease is currently recommended on clinical trials only due to high relapse rates in this setting.

The intensity of pretransplant conditioning regimen is a critical factor in achieving disease control in patients with acute leukemia, and prospective studies have shown the benefit of using myeloablative conditioning (MAC) versus reduced intensity conditioning (RIC) in patients who are eligible to receive either treatment. The antileukemia efficacy and superior relapse-free survival of MAC regimens must be balanced by increased non-relapse mortality and increased incidence of GVHD (Graft versus Host Disease) seen with these regimens, which is caused by increased tissue damage due to MAC regimens that activates alloreactive donor cells. MAC regimens are broadly TBI or chemotherapy based. Based on initial studies reported by Thomas et al., TBI and cyclophosphamide (TBI/Cy) has become the most widely used radiation-based conditioning regimen in patients with ALL (9) and AML (10–14). Non-TBI-containing MAC regimens, mainly based on Busulfan (Bu) and cyclophosphamide/Fludarabine (Bu/Cy and Bu/Flu) regimens, have also been developed and refined for the treatment of AML (12, 15). Because higher doses of BU are associated with sinusoidal obstruction syndrome (SOS) and lower doses are associated with relapse and graft failure, further refinements in drug dosing have been made possible by oral or iv dosing based on pharmacokinetics testing in clinical setting (16). These refinements have resulted in more precise drug delivery, allowing individualized dosing in patients with reduced regimen-related toxicities and lower risk of SOS (Sinusoidal Obstruction syndrome) (17). Currently there is lack of prospective trial data regarding the best MAC regimen in setting of high-risk AML/MDS. The purpose of this review is to summarize role of i) conditioning regimen intensity in disease control in high risk leukemia ii) review data on TBI and BU based MAC regimens in AML/MDS and iii) describe newer developments in safe delivery of higher doses of TBI iv) describe mechanisms of relapse post HCT and possible strategies to overcome this.

## 2 Clinical Outcomes With MAC vs RIC in Patients Undergoing HCT in Morphologic Remission: Results From CIBMTR and EBMT Studies and Importance of Conditioning Intensity for Disease Control

**Figure 1:**
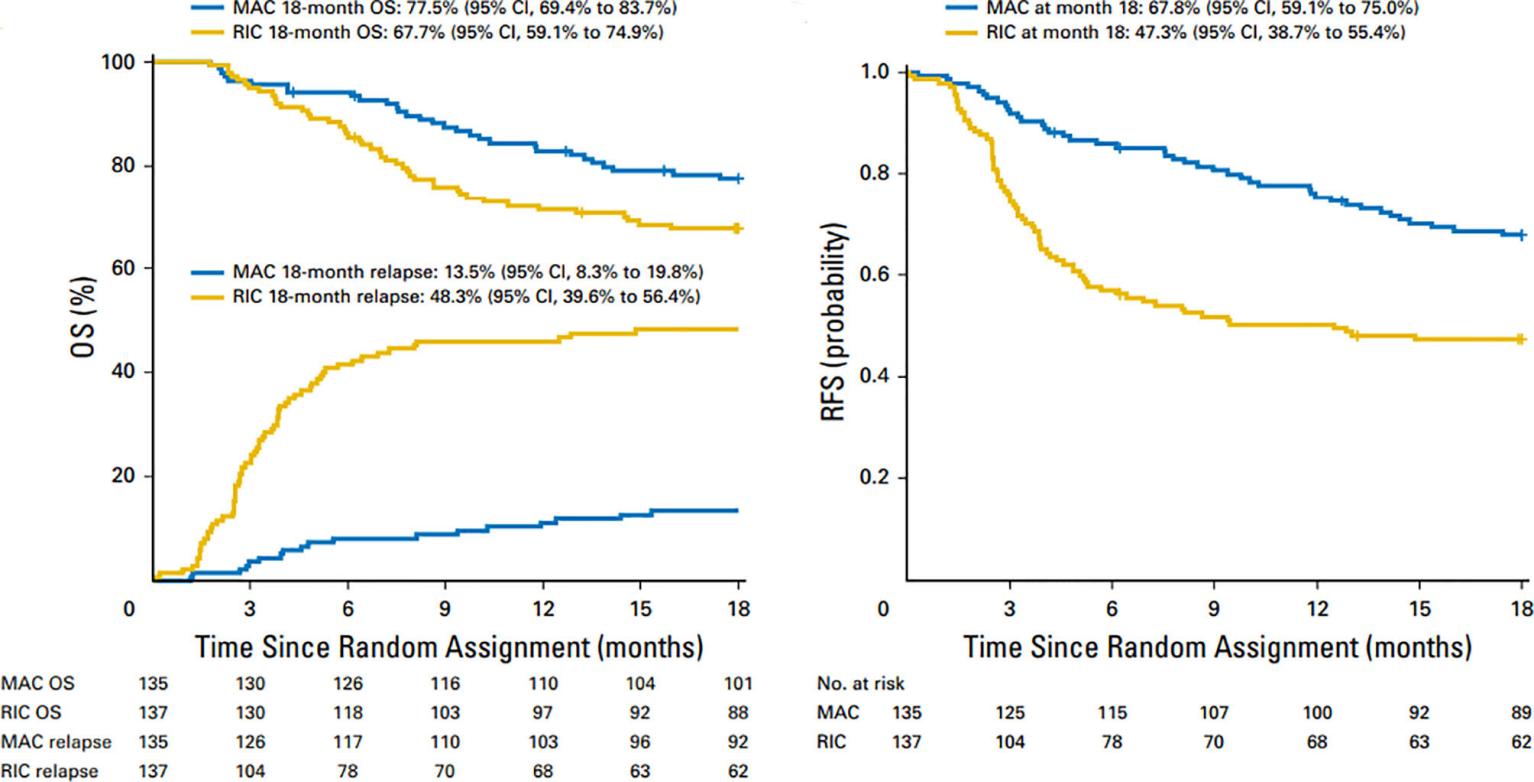
Overall survival and relapse-free survival after 18 months in patients treated with MAC and RIC regimens. Similar OS and higher relapse rates in RIC vs MAC regimens (left panel) and superior RFS with MAC regimens (right panel).

For patients who are in morphologic remission, studies have shown that MAC regimens may yield superior relapse free survival (RFS) compared to reduced intensity conditioning (RIC) regimens. Scott et al. published results of a randomized phase 3 study comparing MAC (n=135) with RIC(n=137) in patients with AML or myelodysplastic syndromes (MDS) with HCT comorbidity index ≤4 who were in morphologic remission pretransplant (18). Patients were randomly assigned to receive either MAC or RIC from HLA matched related or unrelated donor. The primary endpoint was overall survival (OS) at 18 months post-transplant based on intent to treat analysis. Secondary endpoints included relapse-free survival and transplant related mortality. An unexpected high relapse rate was observed in the reduced intensity arm compared with the MAC arm (48.3% vs 13.5%) resulted in early termination of the trial. At 18 months, overall survival (OS) for patients in the RIC arm was 67.7% vs 77.5% (p=0.07) for those assigned to the MAC arm (**Figure 1**). Transplant related mortality (TRM) with RIC was 4.4% vs 15.8% for MAC arm. The conclusion from this randomized BMT CTN study0901 was that RIC resulted in low TRM, but a high rate of relapse compared with MAC regimens, resulting in a statistically significant advantage in relapse-free survival (RFS) with MAC; however, increased TRM with MAC abrogated any benefit noted with this regiment resulting in similar overall survival. On longer term follow up MAC regimens showed superior outcomes compared to RIC with 4 year OS of 65% (MAC) and 49 (RIC; p=.02) (19).

**Figure 2:**
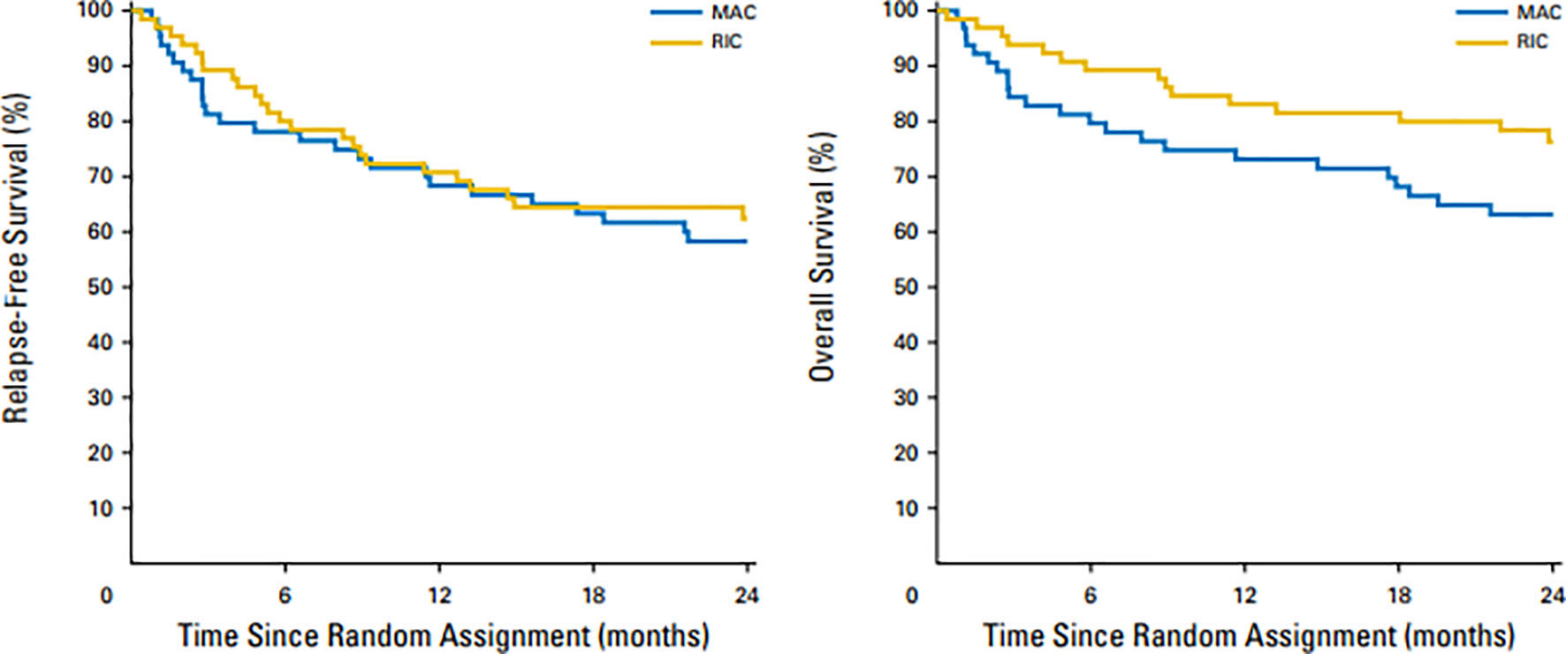
Overall survival and relapse-free survival after 2 years in patients treated with MAC and RIC regimens. Similar OS (left panel) and RFS (right panel) were observed with RIC vs MAC regimens.

The European Society of Blood and Marrow Transplantation (EBMT) simultaneously published the results of a prospective, multicenter, phase 3 study comparing Bu-based RIC with MAC in patients with MDS/AML (RICMAC trial) (20). A total of 129 patients were enrolled from 18 centers. Patients were randomly assigned in a 1:1 ratio and were stratified according to donor, age and blast count. The cumulative incidence of relapse at 2 years was 17% after RIC and 15% after MAC (P = NS) and 2-year RFS and OS were 62% vs 76% and 58% vs 63% respectively, after MAC (P = NS for both) (**Figure 2**). This randomized study sponsored by EBMT confirmed similar OS in AML/MDS patients and, unlike the BMT-CTN 0901 study, did not show superior RFS with MAC regimens. In this study the majority of patients had MDS and <10% patients had secondary AML at time of inclusion in study. It is possible that lower number of high-risk AML patients may have been responsible for lack of RFS benefit seen with MA regimens vs RIC regimens. Intrestingly, the 1 year NRM was similar in MAC and RIC arms (25% vs 17%; p=.29)

In both CIBMTR (Scott et al.) and EBMT (Kroger et al.) studies the MAC regimens were predominantly BU-based, so it is unclear if a TBI-based regimen would have been the superior MAC regimen when used patients with AML/MDS. This question was addressed in two large registry studies in the United States that reported that IV Bu may be superior, or at least noninferior, to TBI-based MAC regimens (21, 22). Both studies were retrospective registry–based with nonrandomized study designs, and had several other additional limitations, including heterogeneity in patient populations, GVHD prophylaxis, and inconsistency of TBI dose that ranged from a nonmyeloablative dose of 550 cGy to as high as 1440cGy.

**Figure 3:**
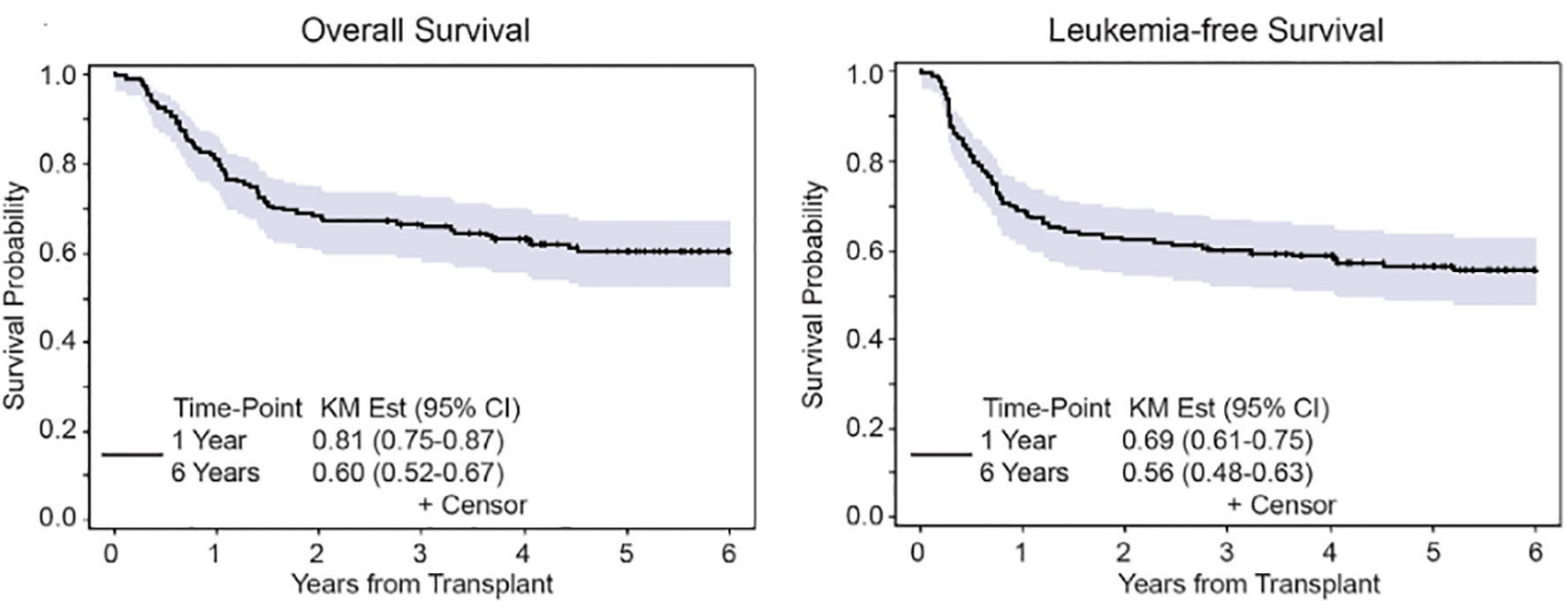
Overall survival and leukemia-free survival after 6 years in AML patients undergoing FTBI based MAC.

We reported our experience in AML patients undergoing allo-HCT using FTBI-based MAC using 1320cGy of radiation in 167 patients, median age 41 years, using either FTBI/Cy (120mg/kg) or etoposide (60mg/kg) (23). Patients undergoing allo-HCT were in first or second remission and received predominantly a PBSC (peripheral blood stem cell) graft from related or unrelated donor. GVHD prophylaxis was with tacrolimus and sirolimus. The 6-year overall survival was 60% and non-relapse mortality was 15% (**Figure 3**). Composite end point of GRFS (GVHD/RFS) was 45% at 1 year and 39% at 2 years. The incidence of late metabolic effects and secondary malignancies was 9.5%. These results compared favorably to IV Bu–based conditioning regimens, without significant long-term toxicity arising from TBI-based regimen, and FTBI-based conditioning regimens are preferred for AML patients eligible for MAC allo-HCT. FTBI-based regimens remain the preferred choice for MAC regimens at our center.

Additional data favoring TBI based MAC regimens over chemotherapy based MA regimens have been reported by investigators and these have shown superior OS/LFS with TBI-based conditioning. Blaise et al. (24) reported their experience in 101 patients with AML in first remission undergoing allo-HCT using a preparatory regimen comprised of Cytoxan 120mg/kg with FTBI vs Bu 16 mg/kg, using HLA matched sibling donors. The outcome for CYTBI was superior in 2 years disease-free survival (DFS) (72% vs 47%; p<0.01), OS (75% vs 51%; p<.02), relapse (14% vs 34%; p<0.04) and transplant mortality (8% vs 27%; p<0.06). Ringden et al. reported results from Nordic bone marrow transplantation group in which 167 patients with leukemia received bone marrow transplant from HLA identical donors with either Bu 16 mg/kg (n=88) or TBI(n=79) with cyclophosphamide 120 mg/kg. The cumulative incidence of venoocclusive disease and hemorrhagic cystitis was higher in the Bu group. In patients with advanced disease, the TRM was significantly higher in the BU group compared to TBI treated patients 62% vs 12%(p=.002). 3-year overall survival was higher in the TBI treated versus Bu treated patient 76% vs 62%; p<.03). The recommended TBI based myeloablative conditioning especially in patients with advanced disease with Bu based conditioning being acceptable in patients with early disease and in those patients where TBI is not feasible (25)

In the recent era, the European Group for Blood and Marrow transplantation performed a retrospective registry-based study comparing outcomes of patients in AML in first or second remission after allogenic transplant from sibling donors who underwent IV Bu/Cy or TBI/Cy-based conditioning. Cumulative incidence of 2-year NRM was 12% in the IV Bu/Cy group and 15% in the Cy/TBI group (P =0.14). The 2-year relapse incidence was 26% and 21% respectively (P= 0.012) in IV BUCy vs TBI/Cy. The LFS (leukemia free survival) rate was 61% after IV Bu/Cy and 64% after Cy/TBI (P= 0.27). In a multivariable analysis, patients who received IV Bu/Cy had lower acute and chronic GVHD, higher RI (relapse incidence), and a trend toward lower NRM. LFS was not statistically different between the two conditioning regimens. In subgroup analysis there was a trend towards better leukemia free survival with Cy/TBI compared with IV Bu in patients with poor cytogenetics and the LFS was 60% versus 43% in favor of MAC arm (26).

These data indicate that in patients with MDS/AML, FTBI-based myeloablative regimens may be preferred for allo-HCT given superior RFS/OS especially in patients with poor risk features, the benefit in low risk AML and MDS patients may be masked by high TRM. Prospective data from randomized trials comparing TBI or Bu based MAC regimens are currently lacking.

The role of TBI-based MAC regimens in patients with acute lymphoblastic leukemia (ALL) undergoing allogenic stem cell transplant is more established. In a meta-analysis performed by Dutta et al. (27) TBI-based regimens were significantly favorable to non-TBI–based conditioning with regards to OS (HR=0.74, 95% CI [0.62, 0.88], 6 studies, 4300 patients), PFS (HR=0.72, 95% CI [0.61, 0.85], 6 studies, 4300 patients), and relapse (RR=0.73, 95% CI [0.61, 0.86], 5 studies, 4664 patients) (27). Based on this and other studies from pediatric literature, in patients with ALL eligible for MAC myeloablative conditioning TBI-based regimens are preferred in ALL patents (28).

## 3 Role of Escalation of Conditioning Intensity and Chemotherapy Pre-Conditioning in Patients With Active Disease

Results of allo-HCT outcomes using standard MAC regimens have been disappointing in patients with active disease. In a large multicenter study reported by the Center for International Blood and Marrow Transplant Research (CIBMTR), in patients with active leukemia (n=2255; 1673 with AML and 582 with ALL) undergoing allogeneic HCT using standard MAC regimens, the 3-year OS was 19% (AML) and 16% (ALL). Day +100 mortality was 59% in AML and 41% in ALL, with relapsed disease being the most common cause for death. In patients with AML predictive factors for poor outcome were: CR-1 ≤ 6 months, presence of circulating blasts, non-sibling donor, poor performance status and adverse cytogenetics. In ALL, primary refractory disease or second relapse, presence of ≥25% marrow blasts, CMV seropositive donor or recipient age more than 10 years correlated with poor survival. Similar outcomes were reported from the Société Française de Greffe de Moelle (SFGM) by Michallet et al. (29) in 379 patients with advanced AML undergoing allo-HCT using TBI-based MAC regimen. In their cohort of 379 patients, 82% had relapsed AML and 18% had primary refractory disease. The median post-transplant follow-up was 7.5 years and the 5-year overall and leukemia free survival was 22% and 20% respectively. In this study the 5-year TRM was reported at 45%. The LFS was better in patients who received transplant in remission and from sibling donors.

Findings from these large registry based studies indicate that standard MAC regimens result in suboptimal OS/LFS in patients with active disease at time of transplant and alternate strategies with intensified conditioning regimen are required in patients with active AML (30).

Investigators using escalation of TBI dose from 1200 cGy to 1575 cGy in AML patients in CR-1 demonstrated decrease in relapse rate from 35% to 12%, although this was associated with increased transplant-related morbidity resulting in similar overall survival (31). A prospective CIBMTR study also showed that in patients with ALL receiving TBI/Cy-based conditioning, the DFS was superior in patients receiving a TBI dose of greater than 1300 (32). The improved patient outcomes after higher doses of TBI could be secondary to higher biological effective doses (BED) delivered to leukemic sites, resulting in low relapse rates and improved DFS and OS. Differences in biologically effective dose can arise from differences in dose rate, number of fractions and dose per fraction (33). Despite evidence of dose-dependent antileukemic activity of TBI, more intense dosing is challenging given increases in toxicity and long-term morbidities (34).

In an attempt to improve HCT outcomes in this high-risk patient population, Brown et al., examined intensification of standard MAC in 40 patients with median age of 33 years, with AML in their first untreated relapse. This study used a combination of fractionated total body irradiation (FTBI) at 1200 cGy with high-dose etoposide (1.8 gm/m2) and cyclophosphamide (150-180 mg/Kg), and demonstrated a 3.5-year DFS of 29%; albeit at the cost of increased transplant related mortality (TRM) of 47% at 3.6 years (35). Long et (36)also reported results of allo-HCT from matched sibling donor in 67 patients with advanced hematologic malignancies using intensified conditioning regimen comprising of FTBI(1320 cGy) with etoposide (60 mg/kg)and cyclophosphamide(60mg/kg). DFS at 3 years was 40% in 32 patients with acute leukemia in 1st relapse or 2nd CR or CML-AP and was 32% in 20 patients with more advanced disease.

**Figure 4:**
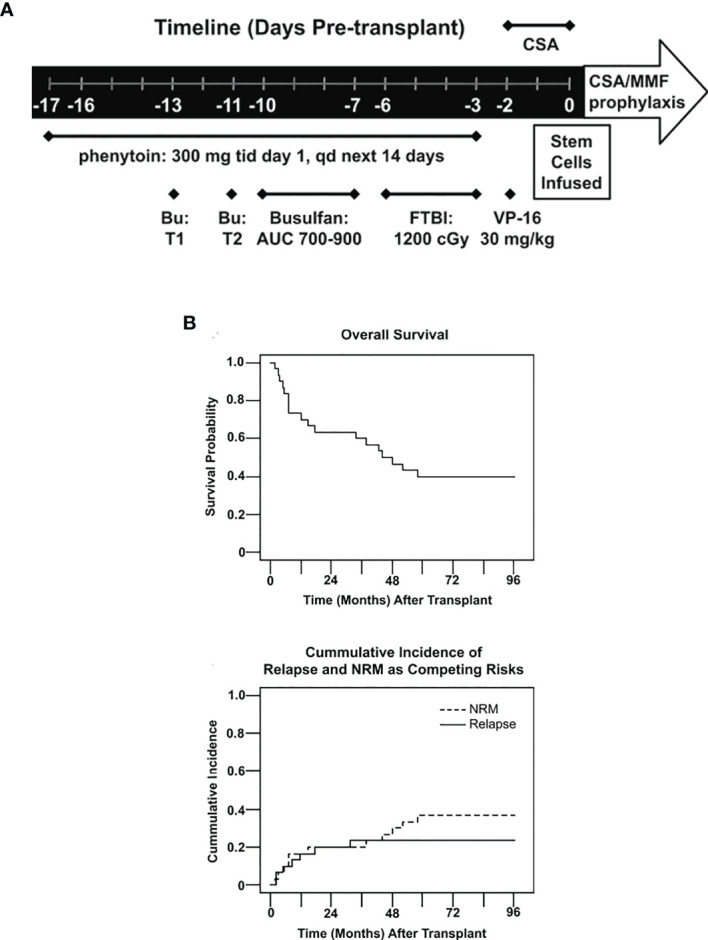
**(A)** Treatment schema. Treatments including dosages are listed under the timeline. BU, bulsulfan, T1, test dose 1, T2, test 2, FTP I, fractionated total body irradiation, CSA, cyclosporine, MMF, mycophenolate mofetil, qd, once daily, tid, three time daily. **(B)** Overall survival probability and Cumulative incidence of relapse and NRM as competing risks.

Our group reported on the results using a modified intensified conditioning regimen using FTBI (1200 cGy) in 10 fractions combined with pharmacokinetic-based IV BU with an AUC of 700–900 µmol/min and etoposide 30 mg/kg using a peripheral blood stem cell graft from HLA matched sibling donors (**Figure 4A**). The regimen was well tolerated, with grade 2 mucositis occurring in 97% patients (Grade 3 in one patient only) along with grade 2–3 skin toxicity in 30% patients. At a median follow-up of 83.7 months, the 5-year overall and DFS was 40% (**Figure 4B**). The cumulative 5-year relapse incidence was 23% and NRM was 37%. This phase 2 prospective study showed that in patients with active leukemia, an intensified conditioning regimen has the potential to cure approximately 40% of patients (37).

Another approach to improve outcomes in patients with active leukemia comprises of chemotherapy prior to HCT conditioning and planned DLI post-transplant. Schmidt at all reported results of prospective study and 75 patients with median age of 52 years with high risk AML/MDS using an approach of sequential chemotherapy followed by RIC allo-HCT (38). Chemotherapy preconditioning comprised of fludarabine 30 mg/m², cytarabine 2 g/m² and amascrine 100 mg/m². (FLAMSA regimen given from days -12 to -9). This was followed by RIC using TBI dose of 400 cGy (day -5), rATG(10 mg/kg for MSD or 20mg/kg for MUD or mismatched donors; days -4 to -2) and 80-120 mg/kg cyclophosphamide(40mg/kg MSD or 60 mg/kg for MUD or mismatched donors;days -4 and -3). GVHD prophylaxis was using cyclosporine and mycophenolate mofetil (MMF). Patient received prophylactic donor lymphocyte infusions (pDLI) if in CR and without evidence of GVHD. With this approach, day +30 bone marrow biopsy showed CR in 88% patients. Overall NRM was 20% at day +100 and 33% at 1 year. The 2-year OS/LFS was 42 and 40% respectively. On multivariate analysis higher CD34 stem cell dose was associated with improved outcome. Results from this trial are impressive given that 80% of the patients had active disease and were ineligible for MAC conditioning. The high NRM of 33% at 1 year was concerning and was attributed to infectious complications and GVHD. In the subsequent multicenter phase 2 study published by the group 103 patients with refractory AML received this normal conditioning regimen and with the median duration of follow-up of 25 months overall survival at 2-year was 40% and leukemia free survival was reported at 37%. In the patients who met criteria for DLI, OS was higher at 87% (39).

Cheong et al. (40) also reported 5-year OS/LFS of 49% and 37% in 56 patients with high risk AML and patients received cytoreductive chemotherapy with either FLAMSA or FLAG ± Ida or CLAG. Majority of the patients received RIC regimen using PBSC graft from related(n=33) or unrelated (n=23) donors. Patient receiving cytoreduction with FLAG or CLAG had lower incidence of NRM and similar relapse rate compared to patients receiving FLAMSA regimen prior to RIC.

Results from these prospective studies show that the approach of using cytoreductive chemotherapy with FLAG/CLAG/FLAMSA, followed by RIC transplant and pDLI in eligible patients can improve outcomes for patients with high risk AML and 2-year OS/LFS comparable to MAC regimens. The results of chemotherapy preconditioning are especially impressive when compared to results of patients undergoing allogenic transplant with active disease using reduced intensity or nonmyeloablative regimens. Wong et al. (41) reported results from MD Anderson cancer Center in 135 patients with active disease in pretransplant bone marrow biopsy and 77% of these patients died with median survival time of 4.9 months. Median progression free survival was 2.9 months. Predictors of poor outcome included low performance status, presence of peripheral blood blasts and high tacrolimus levels post-transplant. To conclude, these studies provide evidence that in patients with high risk AML, novel strategies such as intensification of conditioning chemotherapy, preconditioning with FLAMSA, early taper of IS and planned DLI can help improve 2-3 year OS to 40-45% but high post-transplant relapse and NRM remains a major challenge.

Additional strategies such as graft manipulation and safe escalation of FTBI beyond 1320cGy total marrow irradiation (TMI) and total marrow and lymphoid irradiation (TMLI) in active leukemia using are discussed in next sections.

## 4 Role of Total Marrow Irradiation (TMI) and Total Marrow and Lymphoid Irradiation (TMLI) in Active Leukemia

Conventional TBI delivers beams of radiation of uniform intensity but the body receives a heterogeneous dose due to differences in body thickness, contouring and tissue densities. These variables result in radiation dose that is less than intended to tumor tissue and inadvertent higher doses to normal viscera including heart, liver, lung and the kidneys. The high morbidity associated with standard delivery of FTBI beyond 13.2 Gy has limited the success of this modality. Intensity modulated radiation therapy (IMRT) is a newer technique for radiation delivery initially pioneered in solid tumors wherein the prescribed radiation dose is conformed to target while sparing normal organs. In IMRT the goal is to irradiate bone marrow while sparing other organs (total marrow irradiation,TMI) using either Helical Tomotherapy (HT) (42) or linear accelerator-based intensity modulated TMI (43). Delivering radiation therapy from multiple directions using multiple segmented or modulated beamlets, IMRT allows for greater sculpting of radiation doses to fit the unique shape of each patient’s tumor, optimizing radiation delivery to complex volumes and regions of the body. As a result, the dose to adjacent critical organs is minimized, reducing side effects and allowing for dose escalation to the tumor, thus improving outcomes (44).

Corvo et al. (45) reported results of TMI delivered with HT as boost in 15 patients with high risk leukemia (10 AML and 5 ALL) to intensify dose of radiation beyond FTBI dose of 12 Gy. Patients started conditioning with 12Gy of TBI using conventional linear accelerator delivered as 2 Gy twice daily on days -7, -6 and -5 followed by boost of TMI of 2 Gy in a single fraction on day -4, cyclophosphamide 60 mg/kg with infused on days -3 and -2 followed by allogenic transplant on day 0. The median organ at risk dose reduction with TMI ranged from 30-65% with largest dose reduction noted in brain, larynx, liver, lungs and kidneys. The TMI boost after standard FTBI was well-tolerated and no additional adverse effects noted with radiation boost. All high-risk patients achieved complete remission on day 30 marrow post stem cell transplant. The investigators reported short duration of follow-up (median 310 days) during which 3 patients have died (2 with severe GVHD 1 with infection) and 2 patients relapsed. 12 patients were alive with 10 survivors in remission. This study showed that boost of TMI delivered with HT can be used in high risk patients to improve relapse free survival without significant additional radiation toxicity.

Patel et al. (46) reported on the results of TMI delivered using the linear accelerator to myeloablative chemotherapy regimen prior to allo-HCT. Fourteen patients with high-risk hematologic malignancies were enrolled on a phase 1 study (NCT00988013) and received escalated doses of TMI starting at 3 Gy and gradually escalating up to 12 Gy in combination with IV fludarabine 160 mg/m2 square and BU (AUC 4800 microM*minute). Patient received PBSC graft from related or unrelated donor. GVHD prophylaxis was with tacrolimus and methotrexate. The regimen was well-tolerated, all patients engrafted and nonhematologic toxicity was limited to grade 1-2 oral mucositis. With median follow-up of 37 months the OS/LFS was 50 and 43% in this high-risk patient population. T-cell subset recovery after TMI based allogenic transplant with similar to recovery of incidence post chemotherapy based myeloablative transplant indicating no significant impact on immune reconstitution. In this phase 1 dose escalation study the maximum tolerated dose of TMI in combination with fludarabine and IV BU was 9 Gray. This study showed that TMI delivered through IMRT can be safely added to a backbone of myeloablative chemotherapy regimen potentially allowing patients with advanced hematologic malignancies to benefit from this novel radiation treatment.

Hui et al. (47) reported on the feasibility of using TMI in a phase 1 dose escalation study in high risk patients undergoing Allo-HCT from either umbilical cord blood or sibling donor. Twelve patients (four pediatric and 8 adults) received conditioning with cyclophosphamide and fludarabine in conjunction with image guided radiation to the bone marrow at 15 Gy and 18 Gy in 3 Gy fractions while maintaining TBI dose to vital organs at less than 13.2 Gy. The biological effective dose of TMI delivered to bone marrow was increased by 62% at 15Gy and 96% and 18 Gy compared to standard TBI. At 18 Gy dose level, 6 patients experienced regimen related mortality although DLT defined the graft failure or excessive organ toxicity was not encountered. Subsequently, four additional patients were treated at 15 Gy dose level. The 1- year OS/LFS was 42% and 22% respectively with relapse rate of 36% and NRM of 42%. In the study escalation of TMI dose to 15 Gy in combination of fludarabine and cyclophosphamide was deemed feasible with successful delivery of higher doses of radiation to bone marrow while maintaining the dose delivered to other organs at ≤13.2 Gy. All patients included in the study had active disease or evidence of MRD^+^ disease prior to transplant indicating a very high-risk patient population. The 1-year OS/LFS was deemed promising in this study at 15 Gy dose level with high NRM at higher dose of 18Gy.

Further refinements in TMI has now allowed combination of total lymphoid irradiation (TLI) with TMI, this potentially allows superior engraftment kinetics and extramedullary disease control in patients with advanced hematologic malignancies. For TMI, the gross tumor volume (GTV) comprises of marrow containing bony skeleton. For TLI, the GTV comprises of the entire lymphatic system plus liver and spleen. In ALL, the brain and testes are included given potential for these areas to act as sanctuary sites of disease. This targeted form of radiation delivery selectively targets diseased marrow and lymph nodes while sparing healthy tissue, thereby maximizing the radiation therapeutic index. TMLI allows for precise delivery and increased intensity treatment *via* sculpting radiation to sites with high disease burden or high risk for disease involvement while sparing normal tissue (42, 48–51).

**Figure 5:**
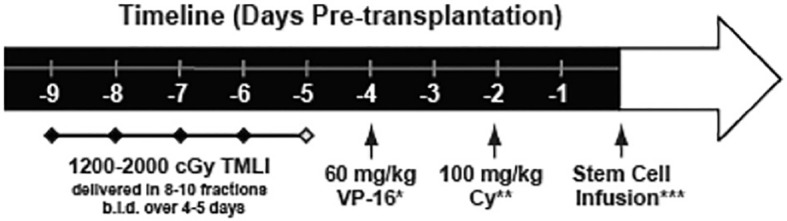
Treatment schema. TMLI was delivered in 8-10 fractions twice daily over 4 to 5 days with total targeted dose ranging from 1200 to 2000 cGy. A window for 1 to 2 days was allowed for stem cell infusion with interval between Cytoxan and stem cell infusion greater than 48 hours.

Based on our groups extensive experience in safely delivering TMLI in patients with advanced hematologic malignancies, we have pioneered the use of novel high intensity allogeneic stem cell transplantation using an escalated dose of radiation using TMLI (up to 20Gy) in combination with high dose chemotherapy in patients with acute leukemia who are treatment refractory or beyond second remission and therefore undergoing transplantation with active disease. We published the results of a phase 1 clinical trial in patients (N=51) with active leukemia using TMLI combined with high-dose cyclophosphamide (100 mg/kg) and etoposide (60 mg/kg) in patients with HLA matched related or unrelated donors (**Figure 5**) (52). This is the largest study reported so far in patients with active leukemia undergoing TMLI based HCT. Patients were eligible to enroll on this clinical trial if they were less than 60 years of age with relapsed or refractory AML or ALL and had active disease at the start of the transplant preoperative regimen. The bone marrow, lymph node chains and testes were escalated up to 2000 cGy with liver, porta hepatis and brain receiving 1200 cGy. Palifermin was administered to reduce the risk of mucositis and GVHD prophylaxis consisted of tacrolimus and sirolimus. Post-transplant maintenance was not part of the planned therapy.

The primary objective of this phase 1 study was to establish the maximum tolerated dose of TMLI and to describe the toxicities at each dose level. Secondary objectives included estimates of NRM, CR rates and overall survival. A total of 51 patients with a median age of 34 years with active disease refractory to salvage chemotherapy were enrolled. Of these, 33 patients had AML, 16 had ALL, and 2 patients had undifferentiated acute leukemia. The majority of patients (42/51) had greater than 10% blasts in the bone marrow at the time of transplantation. Stem cell donors were HLA identical sibling (n=25), matched unrelated (n=5) and mismatched unrelated (n= 21). Cytogenetic risk was intermediate or unfavorable in all patients enrolled. The median WBC count at this time of transplant conditioning was 1.4 (range 0.1–14.9) and median blast percentage in the bone marrow biopsy was 52% (range 5–98). Nine patients had extramedullary disease at the time of transplant.

**Figure 6:**
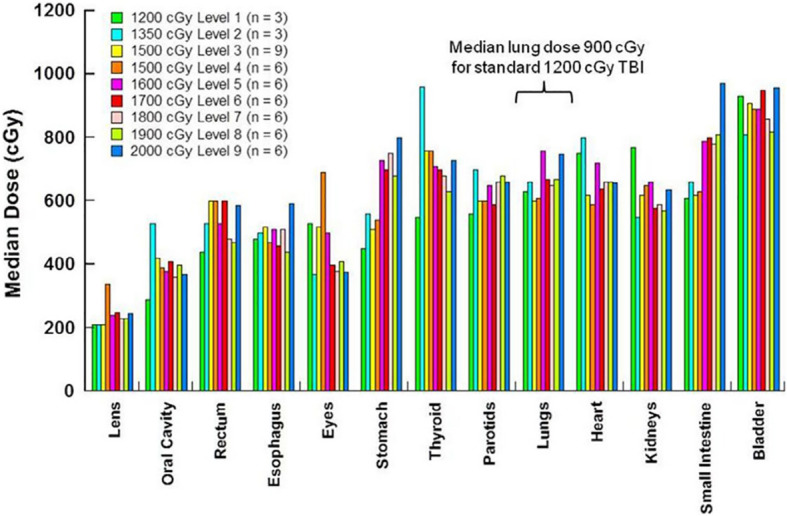
TMLI median organ dose by phase 1 dose levels the dose levels and number of patients at each dose levels are indicated the doses plotted are average for the patient at each dose level.

The primary outcome of this phase 1 clinical trial was to determine the toxicities and maximum tolerated dose with this intensive conditioning regimen using TMLI with Cytoxan and etoposide (**Figure 6**). No early deaths (prior to day 30) were observed among the patients treated, and from day 30 to 100, two deaths occurred (NRM) related to Klebsiella infection and disseminated HHV-6 infection. Bearman toxicities at each dose level are depicted in the **Table 1**. At dose level 4 (1500 cGy), a patient developed grade 3 mucositis attributed to the conditioning regimen; the same patient also developed grade 3 pulmonary toxicity and grade 3 renal toxicity. At the highest dose level of 2000 cGy no DLT’s were observed in the 6-patients treated. Remarkably no patient at any dose level developed veno-occlusive disease.

**Table 1:** Summary of clinical trials with TMI in allo-HCT.

Author, year	Patients	Donor	Conditioning	Outcomes	OS/LFS
Corvo et al. (45)	N=15(AML 10; ALL 5) Active disease 8; CR 7	MSD 7; MUD 8 PBSC graft in all patients	TBI/Cy + TMI boost 2Gy	Median FU 310 days; all engrafted 3 deaths TRM; 2 relapses	80% and 67%
Patel et al. (46)	N=14(AML 9; ALL 2; myeloma 2; CML-AP 1) 5 patients with active disease	MSD 9; MUD 5 PBSC graft in all patients	Flu/Bu + TMI Boost (3 + 3 dose escalation)	Median FU 1126 days, all engrafted 7 deaths; 3 relapse, 4 NRM	50% and 43%
Hui et al. (47)	N=12; ALL 10; AML2 MRD+ 7; active disease 5	MSD 3; UCB9 PBSC 11; BM 1	Flu/Cy +TMI to bone marrow 15 or 18 Gy	1 graft failure;9 deaths (PGF 1; relapse 4; TRM 4	1-year OS/LFS/RR/NRM 42%/22%/36%/42%
Stein et al. (52)	N=51; all active disease. AML 33; ALL 16; other 2	MSD 25; MUD 5; mMUD 21 BM graft 3; PBSC 48	TMLI (dose escalation to 20Gy) with Cy/VP-16; GVHD prophylaxis tacrolimus/sirolimus	All patients engrafted; aGVHD 43%; cGVHD 38%; Day +30 88% CR; Median FU 24 months Deaths 35; relapse 29; NRM 6	1-year OS/RR/NRM 55%/64%/8%

MSD, matched sibling donor; MUD, Matched unrelated donor; PBSC, peripheral blood stem cell; BM, bone marrow; PGF, Primary graft failure.

The study results showed that 51 patients achieve neutrophil recovery at median of 15 days (range 11–23). Platelet engraftment was achieved at a median of 17 days (range 11–77). 7 patients (14%) developed grade 3-4 acute GVHD. The cumulative incidence of acute GVHD day +100 was 43.1%. The median time to onset was 30 days and none of the NRM was attributed to complications of acute GVHD. Chronic GVHD occurred in 26 of 42 patients surviving beyond 100 days. The majority of these had extensive chronic GVHD. Cumulative incidence of chronic GVHD at 1 year was 27.9% and median time to onset was 138 days. 3 patients died of chronic GVHD-related complications beyond day +100. On day 30 post bone marrow biopsy, 45 of 51 patients (88%) achieved morphologic complete remission. All 6 patients at the highest dose level achieved complete remission. With a median duration of follow-up of 24.6 months, 33 patients experienced disease relapse (26 in the bone marrow, 6 extramedullary, and 1 patient with combined marrow and extramedullary relapse). The 1- and 2-year OS was 55.5% and 41.5% respectively. Organ specific toxicity at each dose level is shown in **Table 2**.

**Table 2:** Toxicities by dose level for TMLI study (52).

Organ Assessed	DL1(n=3)1200 cGy		DL2(n=3)1350 cGy		DL3(n=9)1500 cGy		DL4(n=6)1500 cGy			DL5(n=6)1600 cGy		DL6(n=6)1700 cGy			DL7(n-6)1800 cGy		DL8(n=6)1900 cGy		DL9 lead-in(n=6)2000 cGy		
	Grade																				
	1	2	1	2	1	2	1	2	3	1	2	1	2	1		2	1	2	1	2	3
Bladder	0	0	0	0	0	0	1	0	0	0	1	0	0	0		1	1	0	0	0	0
Heart	0	0	0	0	2	1	2	1	0	1	0	1	0	1		1	1	0	0	0	0
CNS	0	0	0	0	0	0	0	0	0	0	0	0	0	0		0	0	0	2	0	0
GI	2	0	3	0	6	0	3	0	0	5	1	1	1	6		0	5	0	4	2	0
Liver	0	0	0	1	0	3	1	1	0	1	0	0	0	0		0	0	1	0	0	0
Lung	0	0	0	0	2	0	0	0	1	0	0	2	0	0		0	1	1	0	0	0
Kidney	0	0	0	0	0	0	1	0	1	0	0	0	1	0		0	0	0	0	0	1
Mouth	2	0	1	0	1	7	2	3	1	3	1	0	0	0		0	1	3	0	1	0

**Figure 7:**
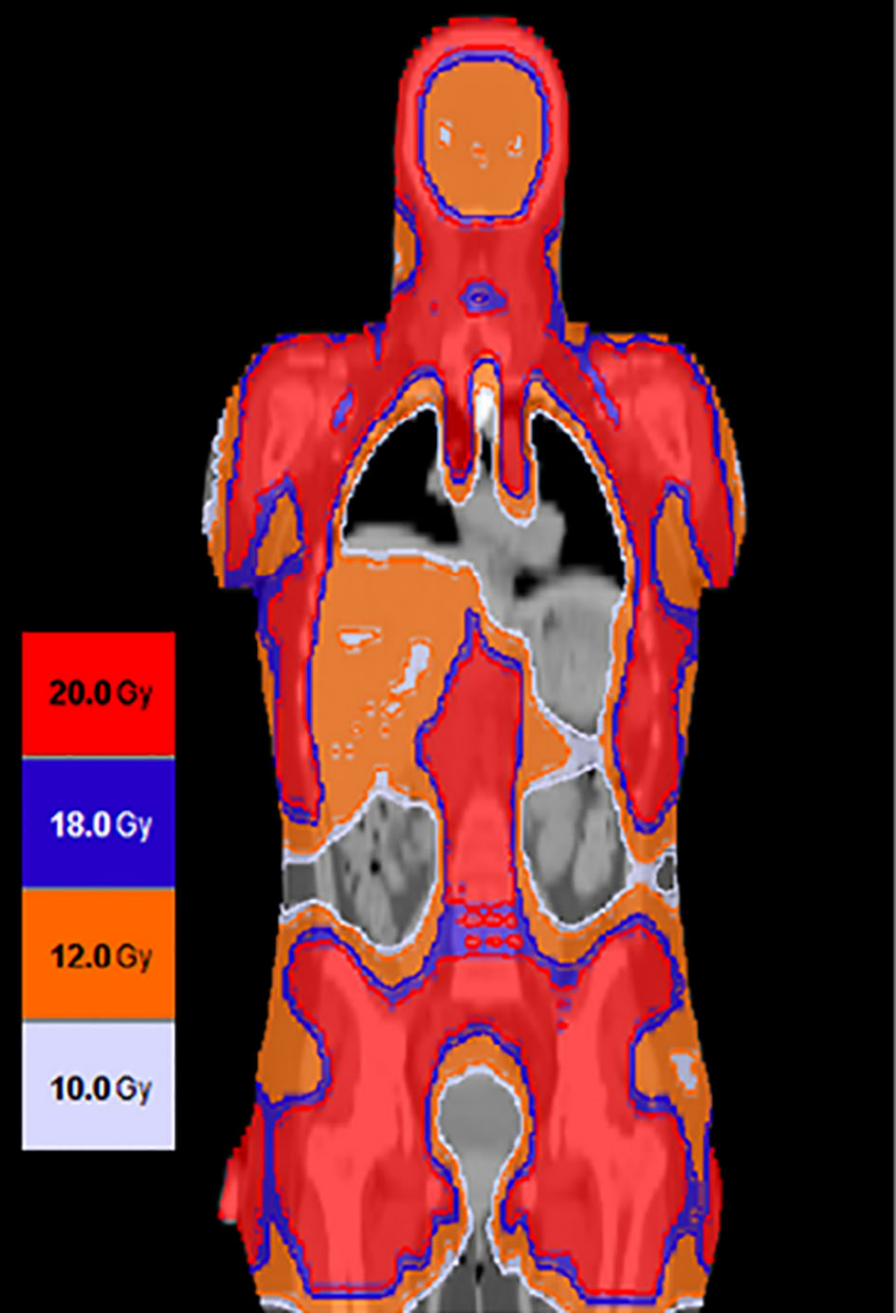
Using targeted dose delivery radiation doses as high as 2000 cGy can be safely delivered to bone and lymph nodes and up to 1200 cGy to liver spleen and brain.

Based on encouraging results from this phase 1 clinical trial, a phase 2 trial is currently underway (NCT02094794) to assess the clinical activity of 2000 cGy of the midline in combination with Cytoxan and etoposide in patients with active leukemia at the time of transplant (**Figure 7**). Using this current regimen, the NRM has been relatively low, and the major cause for transplant failure has been relapse post-transplant.

The results from studies listed above demonstrates that TMI delivered with HT can be successfully delivered to patients without additional toxicity. The radiation dose delivered to bone marrow areas harboring leukemia is similar to TBI while there is significant reduction in radiation dose delivered to organ at risk with TMI-resulting in low TRM. Addition of total lymphoid irradiation to TMI helps with better extramedullary disease control and engraftment kinetics and may potentially reduce incidence of graft failure in high risk leukemia patient population. Despite strategies to escalate conditioning intensity, post-transplant relapse remains major cause of transplant failure and novel strategies need to be implemented to reduce relapse risk beyond conditioning intensification Novel strategies need to be developed in order to reduce relapse post-transplant (53, 54). Mechanisms of post HCT relapse and treatment strategies are discussed in next section.

## 5 Mechanisms of Relapse Post HCT

### 5.1 Residual Disease at Time of Transplant

Presence of measurable residual disease at the time of transplant is considered as high-risk factor for post-transplant relapse in AML across multiple studies (55–57). Strategies are being developed to accurately identify extent of measurable residual disease in pre HCT marrow and novel treatments are being developed to eliminate this clonal population prior to stem cell transplantation (58). The two most commonly validated tools for detection of MRD in pretransplant setting is multiparametric flow cytometric (MFC) analysis and ultra-deep next generation sequencing (NGS) for common myeloid mutations (58). MRD denotes the presence of leukemia cells down to the white blood cell (WBC) level of 1:10^4^ to 1:10^6^ compared to 1:20 in morphologic based assessments. For detection of MRD in AML samples using flow cytometry a comprehensive panel of markers is used including early/stem cell markers(CD34 and CD 117) in addition to myeloid lineage and differentiation markers(CD2, CD7, CD19, CD56) to track aberrant AML blasts. Two separate approaches have been used for assessing MRD by flow cytometry: 1) The leukemia associated with immunophenotype (LAIP) at diagnosis, which tracks baseline immunophenotype and tracks the appearance of abnormal cells surface markers in subsequent samples and 2) the different from normal (DfN) approach, which is based on identification of aberrant differentiation/maturation profiles of samples at follow-up (1, 58). A combination of both approaches is used to diagnose relapse after allo-HCT or chemotherapy using MRD measurement by MFC in clinical use. Molecular MRD assessment is based on RT-PCR based approaches and sequencing for individual DNA molecules. The RT-PCR approach includes Q PCR for producing probes, digital PCR and molecular chimerism analysis. This approach is applicable to approximately 40% of AML patients that harbor 1 more suitable abnormalities that can be detected by RT-PCR. Measurement of MRD using next generation sequencing approaches currently in research development and not ready for use in clinical practice. Current recommendations are to follow molecular MRD in the setting of APL(*PML-RARα*), CBF-AML (RUNX*-RUNX1T1; CBF-MYH11*) and *NPM1-* mutated AML. Due to frequent losses or gains of certain mutations at relapse, current guidelines recommendation against use of mutations such as *FLT3-ITD*, *FLT3-TKD, NRAS*, *KRAS, IDH-1, IDH*-2, *MLL-PTD* and *EV11* as markers of MRD. Preleukemic founder clones such as*DNMT3A*, *ASXL-1* and *TET-2* may persist in the bone marrow even after achievement of morphologic remission hence are not recommended for MRD assessment. Mutations in these genes may also occur in healthy individuals with aging in the form of age-related clonal hematopoiesis (ARCH). Similarly, germline mutations such as *RUNX1, GATA2, CEBPA, DDX41* and *ANKRD26* do not correlate with disease relapse and are not useful for MRD assessment. Thus MRD analysis (MPFC or molecular) provides an objective methodology to establish deeper remission status and to refine prediction of outcomes post allogenic stem cell transplant and identify impending relapse to enable early intervention.

**Figure 8:**
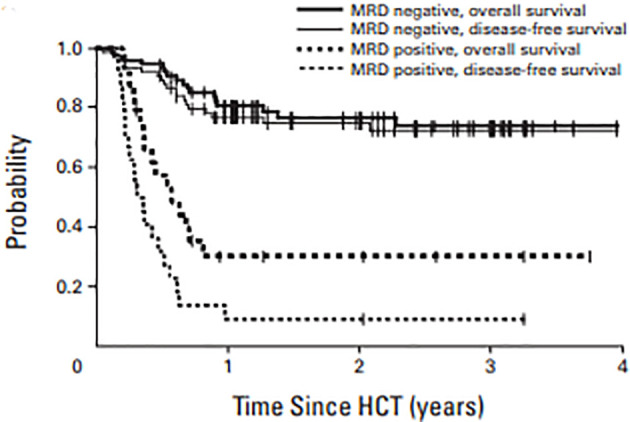
OS and DFS in AML patients in morphologic remission based on MRD by MPFC assessment in pre transplant marrow.

The earliest studies showing benefit of MRD assessment pre-HCT was reported by Walter et al. wherein they retrospectively studied 99 consecutive patients at their center receiving myeloablative transplant for AML in first morphologic remission (56). Ten-color multiparametric flow cytometric (MPFC) was performed on bone marrow aspirates before transplant. MRD was defined as a cell population showing deviation from normal antigen expression patterns compared with normal or regenerating marrow and the level of residual disease was considered as MRD^+^. In this study, 24 patients had MRD^+^ disease prior to transplant as determined by MPFC compared to the rest which were deemed MRD^-^negative. The 2-year estimates of overall survival were 30.2% and 76.6% for MRD^+^ and MRD^-^ patients, respectively (**Figure 8**). Similarly, incidence of relapse was 65% and 17.6%, respectively, in patients who are MRD^+^ vs MRD^-^. In the multivariate model, after adjusting for cytogenetic risk secondary AML, abnormal karyotype and incomplete blood count recovery, MRD^+^ status prior to transplant was associated with increased overall risk of mortality, with a hazard ratio of 4.05 (95% CI 1.9–8.6), and relapse rate of 8.49 (95% CI: 3.67–19.65). This retrospective study for the first time showed that MRD in the pretransplant bone marrow biopsy was an adverse risk factor, with increased risk of relapse and decreased OS.

Usten et al. **l**ooked at the effects of pretransplant MRD^+^ disease by flow cytometry in patients undergoing allogenic stem cell transplant for AML stratified by MAC or RIC regimens (59). They reviewed records of 203 patients, of which 80 received MAC and 123 received RIC regimens. In the MAC arm, 18% had MRD^+^disease compared to 8.1% in the RIC HCT arm. Among RIC patients, MRD^+^ pre HCT was associated with significantly inferior rates relapse (HR 3.8; 95% CI:1.7–8.7), DFS, and OS (HR 3.4; 95% CI: 1.7–7). The authors concluded that in patients with pretransplant MRD^+^status, MAC regimens should be preferred over reduced intensity conditioning.

**Figure 9:**
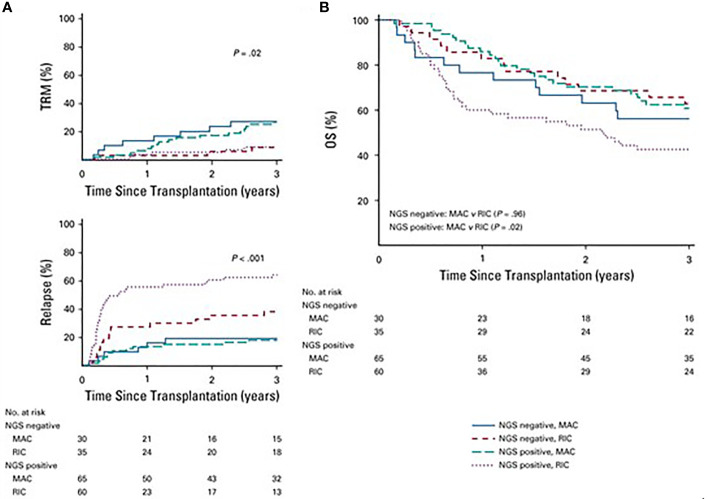
**(A)** Differences in rates of transplant- relates mortality (TRM) based on conditioning intensity and mutational status (P= .02). TRM was significantly hihger in patient who underwent MAC v RIC but there was no difference on the basis mutational satatus (P=8). Patients with RIC had higher relapse rather than MAC (P < .001) with highest risk of relapse in the next-deneration aequencing (NGS) positive patients who received RIC (P < .001). **(B)** In patients who were NGS negative, overall survival (OS) did not differ on the basis of conditioning intensity (3-year OS, 63% RIC v 56% MAC; P= .96). However, in those with detectable mutations, survival was significantly worse in those who received RIC (3-year OS, 43% RIC v 61% MACl P= .02).

More recently, Hourigan et al. used ultra-deep error-corrected sequencing for 13 commonly mutated myeloid genes (*ASXL1*, *DNMT3A*, *FLT3*, *IDH1*, *IDH2*, *JAK2*, *KIT*, *NPM1*, *NRAS*, *RUNX1*, *SF3B1*, *TET2*, and *TP53*) on pretransplant blood in patients with AML treated on a phase 3 clinical trial that randomly assigned patients with AML/MDS in morphologic remission to MAC or RIC. No mutations were detected in 32% of MAC and 37% of RIC recipients, respectively, and the groups had similar 3-year overall survival of 56% versus 63%, respectively In patients with a detectable mutation in the blood by NGS, the incidence of relapse was significantly higher: 67% vs 19% for those with and without detectable mutation, respectively, and 3-year OS as also significantly lower at 43% versus 61%, respectively (**Figure 9**). In multivariate analysis adjusting for disease risk and donor status, RIC was significantly associated with increased risk of relapse (HR 6.38; 95% CI: 3.37–12.1) and decreased OS (HR 1.97; 95% CI: 1.17–3.30) compared with patients receiving MAC (60). The MRD definitions in this manuscript focus on AML – for ALL the reader is referred to additional reviews (61).

The studies listed above confirms that presence of MRD in pretransplant marrow remains a significant risk factor for post-transplant disease relapse. However, relapse risk is lower in patients who receive MAC regimens compared to RIC/NMA regimens. We recommend that in patients with pretransplant MRD^+^disease by MPFC or NGS, consideration should be given for further intensification of conditioning regimen with TMLI based conditioning.

### 5.2 Immune Escape

#### 5.2.1 Genomic Loss of HLA

In patients with hematologic malignancies, alteration in HLA region (somatic mutations) are uncommon at the time of diagnosis (62). This is a crucial factor since donor T-cell mediated graft versus leukemia (GVL) effect depends on HLA molecule expression on leukemic cells. These HLA molecules serve as targets for minor histocompatibility antigens and tumor associated antigens which when incompatible directs allo-reactivity from donor T cells. In the setting of haploidentical stem cell transplantation, loss of heterozygosity in the region of chromosome 6 results in acquired somatic uniparental disomy (aUPD) (63, 64). The genomic alteration consists of loss of a chromosome region that is subsequently replaced by the homologous copy, resulting in acquired homozygosity of that region without actual loss of genomic material (65). Experiments on *ex vivo* cultures of donor T cells with leukemic cells in the setting of HLA loss show that donor T cells are incapable of recognizing leukemic cells thereby providing a mechanism for relapse (66). This has important clinical implications, as donor lymphocyte infusions, which are often used as a strategy for treatment of post-transplant relapse, would be ineffective in this setting. The etiology of acquired aUPD could be increased susceptibility for chromosomal break in leukemic blasts in patients who have been heavily pretreated prior to stem cell transplantation. Patients with large tumor burden prior to transplant and with high tumor heterogeneity are also more likely to carry a clone with HLA loss or have high predisposition to such events, which subsequently leads to relapse post-transplant. This mechanism for leukemia relapse is seen not only in haploidentical but also has been documented in mismatch unrelated and matched unrelated donor transplantation (67). In the setting of fewer donor and recipient incompatibilities, the T-cell allo-reactivity and GVL affect is less pronounced against incompatible HLA haplotypes and is outperformed by immunodominant minor histocompatibility antigens.

Based on this mechanism of relapse the acute leukemia working party(ALWP) of European Society for blood and marrow transplantation (EBMT) has recommended for testing for HLA loss at the time of relapse before proceeding with DLI (68). If there is documented genomic loss of HLA a second allogenic transplant can be done from an alternative donor targeting the remaining HLA haplotype. Oftentimes a second allo-HCT is not feasible in patients who relapse post-transplant due to advanced age or complications from salvage chemotherapy and in this settings non-HLA restricted immunotherapy based clinical trial approaches including bispecific antibodies and chimeric antigen receptor directed treatments may be attempted (69).

#### 5.2.2 Down-Regulation of HLA Class II Molecules

Recent studies have shown that in up to 40% of patients with post HCT relapse, paired patient specimens collected pre HCT in remission and post allo-HCT relapse, the surface expression of HLA class II molecules is diminished resulting in failure of donor T cells to recognize relapse variants (70, 71). RNA sequencing of samples observed at relapse post-transplant revealed down-regulation of major histocompatibility class II genes that was 3–12 times lower than levels seen in pretransplant specimens. Lower HLA class II expression was further confirmed by flow cytometric and immunohistochemical analysis and demonstrated in half of the patients who relapsed in this study. This observation confirmed the importance of interactions between HLA class II molecules and donor CD4 T cells for effective GVL effect, and that this vulnerability can be exploited by leukemia cells to re-emerge post HCT. Genomic HLA loss is proportionate to dose of T cells infused in the graft and is associated with higher likelihood of experiencing relapse. This down-regulation is observed with similar frequencies in both HLA compatible (MUD/MRD) and incompatible transplants (haplo and mMUD). *In vitro* studies have also shown that minor histocompatibility antigens presented by class II molecules far more efficiently compared to HLA class I molecules, implying that in the unrelated donor setting immune reactivity against minor antigens is more potent than against a few incompatible HLA molecules (72). The mechanism for down-regulation of HLA class II genes is not related to somatic mutation in HLA genes or other regulators. Gene expression analysis has revealed significant down-regulation of major histocompatibility class II trans activator *CIITA(MHC2TA)* which is related to hypermethylation/epigenetic silencing of its promoter (73). Interestingly, *in vitro* experiments have confirmed that exposure to high levels of interferon gamma (IFN-γ) can lead to recovery of HLA class II expression on leukemic cells. Exposure of post-transplant leukemic blasts to proinflammatory milieu may lead to recovery of HLA class II expression allowing reconstitution of donor T-cell mediated GVL activity (70, 71). From a clinical standpoint, chronic graft-versus-host disease with chronic inflammatory environment could lead to sustained HLA class II expression resulting in lower leukemia relapse in this clinical setting.

Epigenetic therapies using hypomethylating agent’s (Azacitidine and Decitabine) are commonly used in setting of post-transplant relapse in AML or MDS and may reconstitute donor immune response in post HCT relapse. These agents inhibit DNA methyltransferases thereby altering DNA methylation pattern and causing cell cycle arrest, DNA damage, apoptosis and differentiation. Additionally Azacitidine can stimulate antitumor immunity by up regulation of minor histocompatible antigens (PRAME,NY-ESO1,MAGE-A) on leukemic cells leading to additional graft versus leukemia effect (74). Azacitidine can also increase expression of HLA class I, II molecules and co-stimulatory molecules such as CD80, CD86 on leukemic cells thereby allowing immune recognition of AML blasts (75). Investigators have used combination of azacitidine plus DLI as salvage for post allogenic transplant relapse of AML/MDS. Patients with low disease burden at the time of relapse and dose with longer interval from allogenic transplant relapse tend to respond better with this treatment (76).

Based on the ability of azacitidine to increase expression of some advanced leukemia antigens in order to reduce her cytotoxic T-cell response and to reduce GVHD through induction of Tregs, investigators have used post-transplant maintenance with azacytidine in patients with high risk AML/MDS to reduce relapse. Azacitidine given IV at dose of 32 mg/m2 for 5 days for 4 cycles and high risk AML/MDS patients showed promising 1 year EFS ad OS of 58% and 70% respectively (77). Unfortunately, a subsequently phase 3 study comparing post-transplant 5-azacytidine maintenance versus observation failed to show any improvement in relapse free survival or OS (78). An alternative approach of using azacitidine in patients who experience MRD relapse post-transplant has been more successful in improving transplant outcomes in patient with high risk AML/MDS patients (79). Combination of decitabine and venetoclax in a similar patient population has also been reported to be effective in decreasing relapse rate with 2-year OS of 85% (80).

Histone deacetylase inhibitors such as vorinostat and panobinostat have also been associated with up regulation of major histocompatibility and costimulatory molecules on AML cells through chromatin modification. In a phase 1/2 study in patients with high risk AML who received panobinostat maintenance with DLI(PANOBEST trial), the probability of 2-year OS and RFS was 81% and 75% respectively. The benefit of low relapse rate was offset by higher than expected incidence of chronic graft-versus-host disease in patients receiving DLI indicating that panobinostat does not impair development of peripheral tolerance) chronic GVHD (81).

#### 5.2.3 Up Regulation of T-Cell Inhibitory Ligands

Recent studies have shown that in paired patient samples collected from AML patients at the time of diagnosis and at post-transplant relapse there is increased expression of inhibitory molecules such as PD-L1, CD276/B7–H3 and CD155/PVRL2 and this phenomenon is observed in up to 40% of cases at relapse (82). Overexpression of PD-L1 on leukemic blast impairs T-cell functions and antileukemic responses, which can be partially restored by anti-PD-L1 therapies. Studies have shown that aberrant activation of Jak signaling through 9p24.1 amplification is a potent driver of PD-L1 up regulation in the setting of Hodgkin’s lymphoma. Similar changes are also seen in myeloproliferative neoplasms bearing the Jak-2 V617F point mutation (83). Similarly, Myc oncogenic signaling has been shown to increase the expression of PD-L1 and of CD47(don’t eat me signal) in tumor cells, impairing functioning of T lymphocytes and APCs such as dendritic cells (84). Tumor extrinsic mechanisms such as secretion of IFN-γ can also upregulate PD-L1 on tumor cells. Phenotypic changes are also observed in peripheral blood T-cells at the time of leukemia relapse, and studies have shown significant up regulation of PD-1 receptors in T cells of patients with relapsed leukemia. The expression of inhibitory PD1 receptors on T cells can be mediated by intense stimulation of donor immune system as seen in the setting of haploidentical stem cell transplant and cytokine release syndrome. Expression of exhaustion markers in T cells and relapsing patients with co-expression of inhibitory receptors can synergize in loss of GVL effect resulting in post-transplant relapses (85). This exhausted T-cell phenotype is particularly seen in the bone marrow niche where donor T-cell and leukemia interactions occur for clearance of leukemic blasts (85).

Besides down-regulation of HLA class II molecules and expression of T-cell inhibitory ligands, changes in the microenvironment can also contribute to post-transplant relapses. Presence of an immunosuppressive microenvironment as seen by expression of cytokines such as IL-10 and transforming growth factor beta (TGF-β). This provides a permissive environment favorable to relapse. Changes in the microenvironment associated with decreased production of IL-15 and IFN-γ from myeloid cells can favor leukemia relapse, as IL-15 secretion expands and activates effector T and NK cell antileukemic responses. The FLT-ITD mutation in AML blasts is associated with decreased IL-15 secretion which may be a potential mechanism of relapse in this AML subtype (86, 87).

Immune checkpoint inhibition by monoclonal antibody therapy targeting PD-1/PD-L1 and CTLA-_4_/B7 pathway is a potentially attractive strategy to enhance alloreactive T-cell function in the setting of post-transplant relapse. Studies have shown that the phenotypic features of T cells in patients with relapse post allo-HCT correlates with exhaustion features. This exhausted T cell phenotype is particularly seen in the patient’s bone marrow where T-cell and AML interactions are expected to occur in association with skewed T-cell receptor repertoire (85). Clinical trials reporting immune checkpoint blockade with anti-CTL A4 antibody ipilimumab have shown promising initial results (88) and studies using PD1 inhibitors have shown efficacy in setting of Hodgkin’s lymphoma (89). Caution is needed in these approaches as post-transplant treatment with checkpoint inhibitors is associated with significant risk of reactivating graft-versus-host disease and immune related adverse events (90). A phase 2 study exploring the combination of anti-PD1 monoclonal antibody nivolumab and azacitidine and relapsed AML reported an overall response rate of 33% and several sites studies are ongoing to assess the efficacy of hypomethylating agent and checkpoint blockade combinations in post-transplant relapse (91).

## 6 Future Directions

In patients with active leukemia, MAC regimens that use standard doses of radiation result in increased post HCT relapse and suboptimal clinical outcomes. Adoption of new techniques to deliver radiation using total marrow and lymphoid irradiation has allowed safe delivery of higher doses of radiation up to 2000 cGy to bone marrow areas while sparing normal tissues, allowing better disease control and GFRS outcomes. However, relapse of primary disease remains the prime cause of transplant failure. Incorporation of adoptive immunotherapy using sequential infusion of Treg: Tcons along with CD34 HPCs using the approach pioneered by Perugia group may help reduce relapse rates while keeping GVHD rates low. In a prospective study recently published by the group, using a conditioning regimen of TBI/TMLI fludarabine/thiotepa/cyclophosphamide, infusion of Tregs : Tcons in a ratio of 2:1 in haploidentical setting in patients with AML in remission, without any post-transplant immunosuppression, led to cGVHD/RFS (CRFS) of 75% (92). This approach is attractive in the active disease setting as no post HCT immunosuppression allows early immune reconstitution that may have a beneficial effect on relapse rates. Similarly, depletion of naïve T cells (Tn) from a PBSC graft while preserving memory T cells (Tmem), Treg and NK cell components has been successfully used in the setting of matched donor and haploidentical setting with promising early results (93, 94). Other strategies to decrease relapse rates post HCT in a high-risk setting include maintenance therapy with targeted agents, planned hypomethylating agent therapy with DLI, MRD monitoring, and immune checkpoint blockage post HCT (95, 96).

## Author Contributions

ASa wrote the review and ASt critically reviewed and edited the manuscript. All authors contributed to the article and approved the submitted version.

## Funding

Funding for IST from BMS. The work was partially supported by the National Institutes of Health under R01CA154491 (S. H.). The funders were not involved in the study design, collection, analysis, interpretation of data, the writing of this article or the decision to submit it for publication.

## Conflict of Interest

The authors declare that the research was conducted in the absence of any commercial or financial relationships that could be construed as a potential conflict of interest.

## Publisher’s Note

All claims expressed in this article are solely those of the authors and do not necessarily represent those of their affiliated organizations, or those of the publisher, the editors and the reviewers. Any product that may be evaluated in this article, or claim that may be made by its manufacturer, is not guaranteed or endorsed by the publisher.
